# Secretory production of tetrameric native full-length streptavidin with thermostability using *Streptomyces lividans* as a host

**DOI:** 10.1186/s12934-014-0188-y

**Published:** 2015-01-13

**Authors:** Shuhei Noda, Takuya Matsumoto, Tsutomu Tanaka, Akihiko Kondo

**Affiliations:** Biomass Engineering Program, RIKEN, 1-7-22 Suehiro-cho, Tsurumi-ku, Yokohama, Kanagawa 230-0045 Japan; Organization of Advanced Science and Technology, Kobe University, 1-1 Rokkodai, Nada, Kobe, 657-8501 Japan; Department of Chemical Science and Engineering, Graduate School of Engineering, Kobe University, 1-1 Rokkodai, Nada, Kobe, 657-8501 Japan

**Keywords:** *Streptomyces*, Streptavidin, Secretory production, Thermostability

## Abstract

**Background:**

Streptavidin is a tetrameric protein derived from *Streptomyces avidinii*, and has tight and specific biotin binding affinity. Applications of the streptavidin-biotin system have been widely studied. Streptavidin is generally produced using protein expression in *Escherichia coli*. In the present study, the secretory production of streptavidin was carried out using *Streptomyces lividans* as a host.

**Results:**

In this study, we used the gene encoding native full-length streptavidin, whereas the core region is generally used for streptavidin production in *E. coli*. Tetrameric streptavidin composed of native full-length streptavidin monomers was successfully secreted in the culture supernatant of *S. lividans* transformants, and had specific biotin binding affinity as strong as streptavidin produced by *E. coli*. The amount of Sav using *S. lividans* was about 9 times higher than using *E. coli*. Surprisingly, streptavidin produced by *S. lividans* exhibited affinity to biotin after boiling, despite the fact that tetrameric streptavidin is known to lose its biotin binding ability after brief boiling.

**Conclusion:**

We successfully produced a large amount of tetrameric streptavidin as a secretory-form protein with unique thermotolerance.

**Electronic supplementary material:**

The online version of this article (doi:10.1186/s12934-014-0188-y) contains supplementary material, which is available to authorized users.

## Background

**Figure 1:**
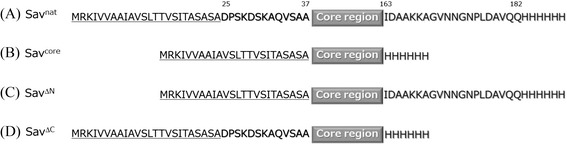
**Sav variants constructed in this study. (A)** Sav^nat^, native full-length Sav; **(B)** Sav^core^, Sav composed of the core region; **(C)** Sav^ΔN^, N-terminal region truncated Sav; **(D)** Sav^ΔC^, C-terminal region truncated Sav. Underline indicates original signal peptides of Sav.

Streptavidin (Sav) is a tetrameric protein produced by *Streptomyces avidinii*, and has tight and specific biotin binding affinity with a dissociation constant of about 10^−15^ M [1-3]. The Sav-biotin system is widely used for biomolecule labeling, purification, immobilization, and more sophisticated biotechnology applications [4-6]. Sav is usually produced using recombinant *Escherichia coli* carrying the gene encoding the Sav core region (Sav^core^) [7-10]. Sav^core^ consists of the residues Glu-14 to Ala-138 of native full-length Sav (Sav^nat^), and is known to have resistance against further degradation by various proteases (Figure 1) [2,11]. The molecular weight of Sav^core^ is 53 kDa, whereas that of Sav^nat^ is 66 kDa. Various Sav variants, such as Sav with higher affinity to biotin, have been produced using Sav^core^ as the base [3,12]; however, there are few reports about the production of Sav^nat^ or its variants, and the N- and C-terminus regions have been recognized as useless parts in Sav.

*Streptomyces* are gram-positive, filamentous soil bacteria known for their ability to secrete heterologous proteins in culture supernatants [13-15]. *Streptomyces lividans* is the most versatile host among this genus for the production of useful proteins. The secretory production of useful proteins is an important tool for protein production, and is industrially effective due to the simple purification procedures involved without refolding or protein extraction from the cell. There are a large number of reports concerning the secretory production of useful proteins using *S. lividans* as the host. Pozidis et al. successfully used it to produce a biopharmaceutical, mTNFa, derived from mouse [14]. Zhang et al. reported the secretory production of human interleukin-4 receptor [15]. Thus, *S. lividans* has great ability to express heterologous proteins, and has attracted attention as an industrial host for protein production [12]. Although the secretory production of various complex proteins, including mammalian proteins, using *S. lividans* has been demonstrated, there are few reports concerning the secretory production of multimeric proteins, such as dimers or tetramers.

In the present study, we carried out the secretory production of tetrameric Sav^nat^, which retained affinity to biotin, using recombinant *S. lividans* as the host. We previously reported the secretory production of various useful proteins using *S. lividans* as a host [16]. Using this system, we tried to produce Sav^nat^ and successfully demonstrated that *S. lividans* can be used for the secretory production of active-form tetrameric Sav. This result strongly suggests that *S. lividans* can be used for the secretory production of other useful multimeric proteins. In addition, to investigate the contribution of the N- and C-terminal regions of Sav^nat^ in Sav production using *S. lividans*, three truncated Sav variants, Sav^ΔN^, Sav^ΔC^, and Sav^core^, were constructed. Using these variants, we demonstrated that the N- and C-terminal regions of Sav were of significance in Sav production using *S. lividans*.

## Results and discussion

### The secretory production of Sav^nat^ using *S. lividans* and evaluation of biotin binding ability

**Figure 2:**
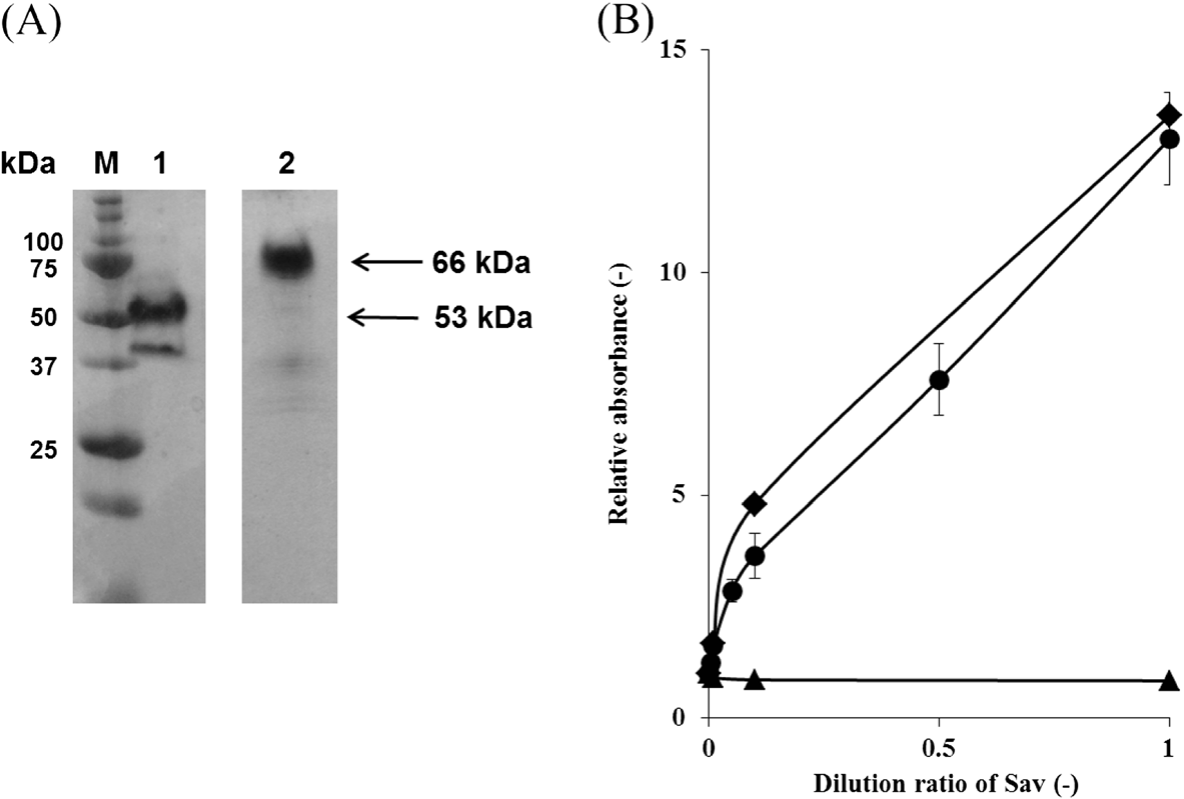
**Sav standard sample and Sav** ^**nat**^ **produced in this study were evaluated using SDS-PAGE and biotin-avidin conjugation assay using biotin coated plates. (A)** SDS-PAGE of Sav standard sample (Lane 1) and purified Sav^nat^ produced by *S. lividans* (Lane 2). **(B)** Biotin-avidin conjugation assays of Sav standard sample (diamonds), purified Sav^nat^ (circles), and BSA as a control (triangles).

Today, Sav is commercially produced using an *E. coli* expression system. Although Sav is a secretory-form protein originating from a species of *Streptomyces*, *S. avidinii*, its production using recombinant *Streptomyces* hasn’t been demonstrated. In the present report, we carried out secretory production of tetrameric Sav using *S. lividans* as the host strain. In order to produce Sav as a secretory-form protein using *S. lividans*, we constructed a vector for Sav expression. Although the synthetic gene of Sav^core^ is usually used for Sav production in *E. coli* systems, we adopted the original full-length gene of Sav^nat^ derived from *S. avidinii*, which has high GC-content similar to the genome of *S. lividans*. Sav^nat^ involves additional amino acid residues at the N- and C-termini compared to Sav^core^ (Figure 1). Figure 2(A) shows SDS-PAGE analysis of a standard sample of Sav (calculated MW, 53 kDa) and the purified Sav^nat^ produced by *S. lividans* (calculated MW, 66 kDa). Here, we evaluated biotin binding ability of Sav^nat^ produced by recombinant *S. lividans*. After immobilization of Sav^nat^ on a biotin-coated polystyrene plate, the unbound molecules were washed out, and then the biotin binding ability was assayed using biotin-HRP. Figure 2(B) shows the assay results for Sav^nat^ produced by *S. lividans* and a standard sample of Sav. Similar curves were observed between Sav^nat^ produced by *S. lividans* and the standard sample of Sav, whereas no corresponding curve was found when using BSA as a control. We thus successfully obtained active-form tetrameric Sav^nat^ using the secretory production system of *S. lividans*.

One of the advantages of secretory production system using *S. lividans* is the productivity of the protein of interest. There are a lot of reports concerning the secretory production system using *S. lividans* as the host [13-16]. The secretory production of useful protein is one of practical ways for industrial protein production due to the simple purification procedures that proteins could be purified directly from the culture broth [14].

Here, to evaluate the utility of our secretory production system in Sav production, the Sav productivity using *S. lividans* was compared to that of using *E. coli*. 0.61 mg of Sav^Eco^ was purified from the 100 mL culture broth, whereas the amount of purified Sav^nat^ using *S. lividans* as the host reached 3.1 mg from the 100 mL culture broth. In order to achieve the further Sav productivity, the signal peptide derived from phospholipase D from *S. cinnamoneus* (*pld* signal peptide) was fused in front of the gene encoding Sav^nat^ [16], meaning that Pld signal peptide existed in the N-terminus of the original signal peptide of Sav, and *S. lividans*/psSav^nat^ was created. As the result, 5.6 mg of Sav^nat^ was purified from the 100 mL culture broth using *S. lividans*/psSav^nat^. Thus, the productivity of Sav using *S. lividans* was 9.2 times higher than that of using *E.coli*. There are some reports concerning Sav production using *E. coli* expression system. The amount of purified full-length Sav from 100 mL of culture broth reached 20 mg [9]. However, in that expression system, fermentor was used for Sav production, and the culture condition needed to be strictly controlled. In this study, Sav and the variants can be expressed and purified by using our simple and conventional procedure. This has a great benefit that we can rapidly produce and evaluate various types of Sav mutants. Recently, Nogueira et al. demonstrated full-length Sav production using the secretory production system using *Pichia pastoris* [17], and this system may be also applied to Sav variants production. Thus, secretory production can be one of promising tools to produce Sav variants, whereas the large amount of Sav could be obtained using large scale fermentation.

### Production of truncated Sav variants and evaluation of the biotin binding ability

There are many reports concerning Sav production using *E. coli* as the host [7,9,10,18]. In these studies, Sav is produced as Sav^core^, which consists of the residues Glu-14 to Ala-138 of Sav^nat^_,_ and Sav^core^ is famous for having resistance to further degradation by many kinds of proteases [2]. However, there are few reports concerning Sav^nat^ production, and the N- and C-terminal regions are not usually regarded as important in Sav production using *E. coli*. Here, we hypothesized that, in the case of secretory-form Sav production using *S. lividans*, the N- and C-terminal regions in Sav are significant.

**Figure 3:**
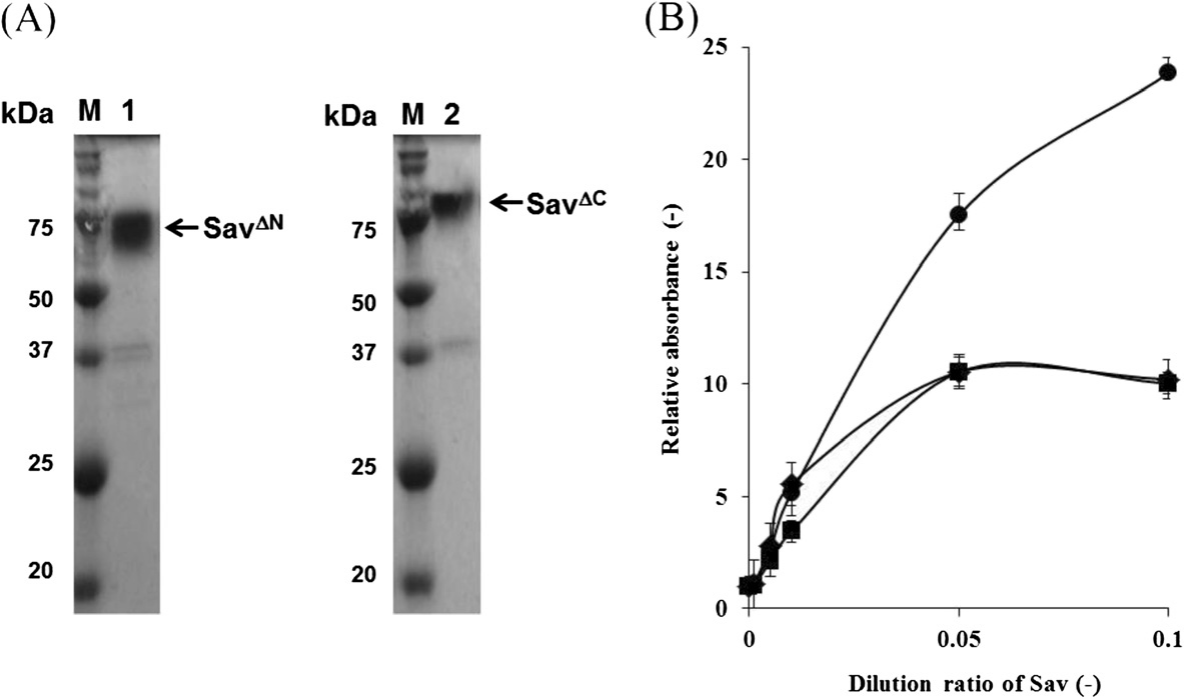
**Sav** ^**nat**^ **, Sav** ^**ΔN**^ **and Sav** ^**ΔC**^ **produced in this study were evaluated using SDS-PAGE and biotin-avidin conjugation assay using nickel coated plates. (A)** SDS-PAGE of purified Sav variants (Lane 1, Sav^ΔN^; Lane 2, Sav^ΔC^). **(B)** Biotin-avidin conjugation assays of each Sav and the variant, Sav^nat^ (circles), Sav^ΔC^ (diamonds), and Sav^ΔN^ (squares).

To investigate the role of the N- and C-terminal regions of Sav^nat^, we tried to construct three Sav variants using *S. lividans*: Sav^core^, Sav^ΔN^, and Sav^ΔC^ (Figure 1). *S. lividans*/Sav^core^, *S. lividans*/Sav^ΔN^, and *S. lividans*/Sav^ΔC^ were successfully created. After cultivation, Sav^ΔN^ and Sav^ΔC^ were purified from the culture supernatants of *S. lividans* transformants; however, not enough Sav_core_ was obtained for purification. Figure 3(A) shows SDS PAGE of purified Sav^ΔN^, and Sav^ΔC^ using affinity column chromatography. We then evaluated their biotin binding ability. Figure 3(B) shows biotin binding assay results for each Sav variant. Sav^ΔN^ and Sav^ΔC^ showed lower biotin binding ability, compared to Sav^nat^ (Figure 3(B)).

Some secretory proteins derived from *Streptomyces* are expressed as pro-proteins with a pro-domain. The pro-domain is known to play an important role and to promote correct folding and translocation of the protein into the culture supernatant. In secretory production of transglutaminase (MTG), the pro-domain of MTG is a significant factor governing its specific activity and productivity [19]. In the present study, Sav^core^ couldn’t be obtained by using *S. lividans* secretory system although Sav is usually produced as Sav^core^ in *E.coli* system. In addition, the biotin binding affinity of Sav^ΔN^ and Sav^ΔC^ were decreased, compared to that of Sav^nat^ (Figure 3(B)). Our current research might indicate that the N- and C-terminal regions of Sav are of significance to retain the activity, as well as the pro-domain of MTG.

### Evaluation of the thermostability of Sav produced by *S. lividans*

Sav is a powerful tool in biotechnology and has been applied in various systems such as biomolecule labeling and immobilization of proteins or small molecules [4-6]. However, tetrameric Sav can usually be dissociated into monomeric Sav by simple boiling [3,9,12], and thus can be utilized only under mild conditions. Therefore, the construction of thermostable Sav can expand its potential applications. For instance, assembling biomass degradation enzymes from extreme thermophilic microbes on thermostable Sav will allow for artificial cellulosomes capable of reacting with high temperatures [20,21]. There are some reports concerning the importance of the thermostability of Sav or Sav mutants, and Chivers et al. successfully constructed a thermostable Sav mutant with the double mutation S52G/R53D [12].

**Figure 4:**
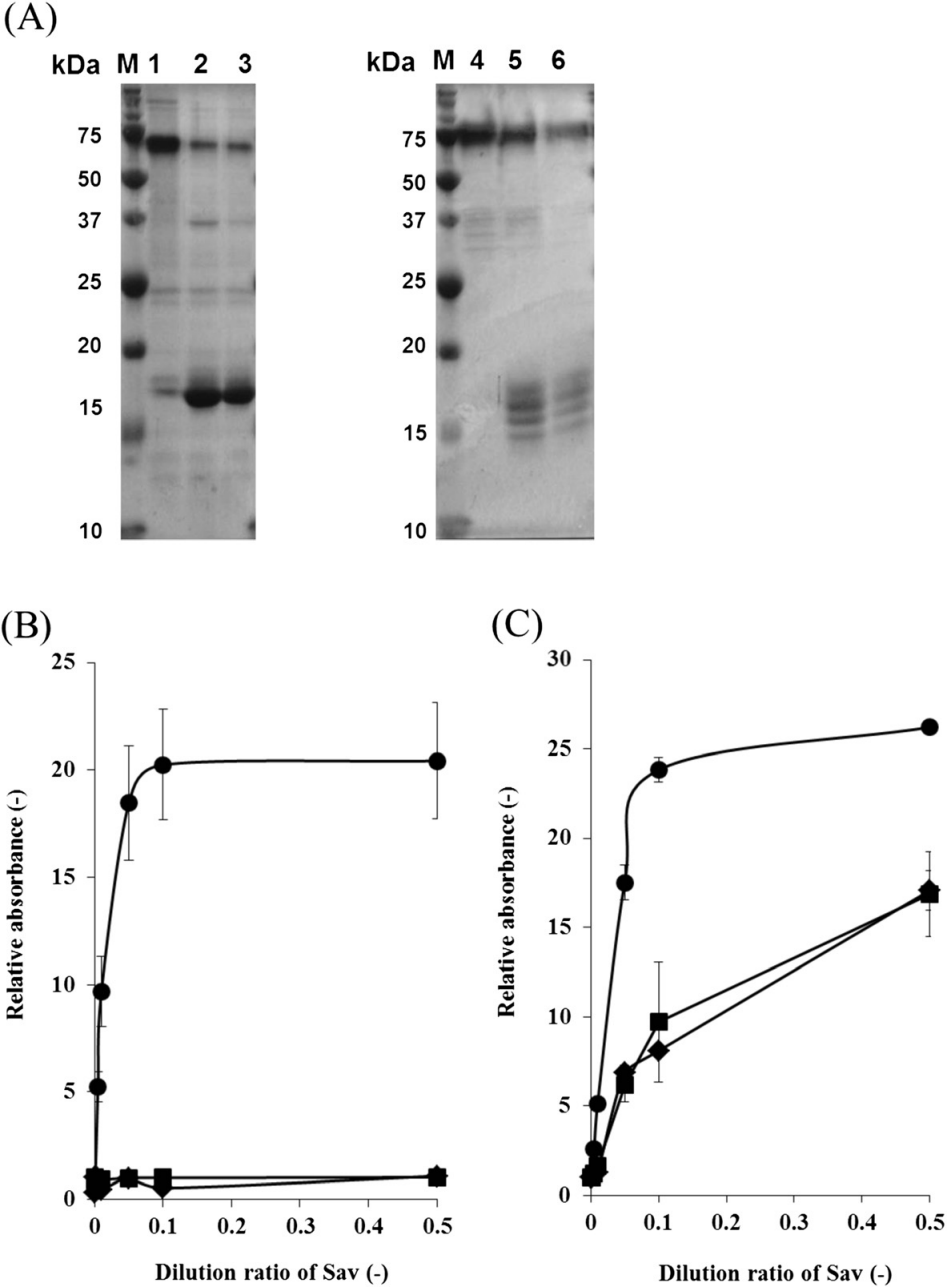
**Thermostability of Sav** ^**nat**^ **produced by using** ***S. lividans*** **in this study was evaluated, and compared to that of Sav** ^**Eco**^ **produced by using** ***E.coli*** **. (A)** SDS-PAGE of Sav^Eco^ produced by *E. coli* (lanes 1–3) and purified Sav^nat^ produced by *S. lividans* (lanes 4–6) after boiling at 100°C for 0 min (lanes 1, 4), 10 min (lanes 2, 5), and 60 min (lanes 3, 6). **(B)** Biotin-avidin conjugation assays of Sav^Eco^ after boiling at 100°C for 0 min (circles), 10 min (squares), and 60 min (diamonds). **(C)** Biotin-avidin conjugation assays of Sav^nat^ after boiling at 100°C for 0 min (circles), 10 min (squares), and 60 min (diamonds).

In this study, we carried out the SDS-PAGE analysis of Sav^nat^ produced by *S. lividans* after boiling at 100°C for 60 min. Surprisingly, we found thermotolerance in this Sav^nat^, although tetrameric Sav is generally known to dissociate into monomers and lose its biotin binding affinity after brief boiling. The molecular weight of Sav^nat^ monomer is 16.5 kDa whereas that of Sav^core^ is 13.2 kDa. Figure 4(A) shows the SDS-PAGE analysis of the thermostability of Sav^Eco^ produced by *E. coli* and Sav^nat^ produced by *S. lividans*. Each Sav was incubated at 100°C for 60 min. As shown in Figure 4(A), Sav^Eco^ was almost completely dissociated after 5 min of incubation (Figure 4(A), lanes 1–3), whereas there was no complete dissociation of Sav^nat^ produced by *S. lividans* (Figure 4(A) lanes 4–6). According to previous reports concerning thermostability of streptavidin, streptavidin-biotin complex indicates high thermostability compared to streptavidin [22,23]. Here, we tried to quantify the amount of biotin binding to Sav^nat^ by HABA assay. However, no biotin was detected in purified Sav^nat^, and thermostability of couldn’t be attributed to biotin binding. We also evaluated the biotin binding ability of each Sav. Figure 4(B) and (C) show the results of assays for biotin binding for each Sav after incubating at 100°C. In the case of Sav^Eco^ produced by *E. coli*, the biotin binding ability was inactivated after only a 5-min incubation (Figure 4(B)). In the case of Sav^nat^ produced by *S. lividans*, although its biotin binding ability decreased, it retained much of its binding ability after boiling for 60 min. These results were consistent with those obtained from SDS analysis (Figure 4(A)). Although we also tested the thermostability of Sav^ΔN^ and Sav^ΔC^, there was no complete dissociation of each Sav variant as well as Sav^nat^ (data not shown). These results may imply that N- and C-terminal regions of Sav^nat^ have no effect of the thermostability.

## Conclusion

In the present study, Sav production using *S. lividans* as a host was successfully achieved. Sav was produced in its tetrameric form and had tight and specific biotin binding affinity. The Sav productivity using *S. lividans* system was 9.2 fold higher than *E. coli* system. In secretory production of Sav using *S. lividans*, the N- and C-terminal regions of Sav were necessary for correct folding and the Sav productivity. These results indicate that our protein secretion system may be used for the secretion of other multimeric proteins. In addition, Sav produced by *S. lividans* exhibited thermostability, and its biotin binding affinity was retained after boiling. This thermotolerant Sav has many potential applications in biotechnology.

## Methods

### Plasmid construction, transformation, and cultivation

*Escherichia coli* NovaBlue {*endA1 hsdR17*(r_*K12*_^−^ m_*K12*_^+^) *supE44 thi-I gyrA96 relA1 lac rec*A1/F’[*proAB*^+^*lacI*^q^ ZΔM15::Tn10(Tet^r^)]} (Novagen, Inc., Madison, WI, USA), used to construct plasmids, was grown in Luria-Bertani (LB) medium containing 40 μg/ml kanamycin at 37°C. The vectors for protein expression using *S. lividans* as a host were constructed as follows. The strains and the plasmids used in this study are summarized in Table 1. Polymerase chain reaction (PCR) was carried out using PrimeSTAR HS (TAKARA BIO, Shiga, Japan). The gene fragment encoding Sav^nat^ was amplified by PCR using *S. avidinii* (NBRC13429) as a template with the corresponding primers (Table 1). The Sav^nat^ fragment was introduced into the *Nde*I and *Hin*dIII sites of pTONA4. The resultant plasmid was called pTONA4- Sav^nat^. The gene fragment encoding Sav^ΔN^ or Sav^ΔC^ was amplified by PCR using pTONA4-Sav^nat^ as a template with the corresponding primers (Table 1), respectively. The Sav^ΔN^ or Sav^ΔC^ fragments with the signal peptide sequences of Sav, Sav-sig_F, and Sav-sig_R, were introduced into the *Nde*I and *Hin*dIII sites of pTONA4, respectively. The resultant plasmids were called pTONA4-Sav^ΔN^ and pTONA4-Sav^ΔC^, respectively. The gene fragment encoding Sav^core^ or Sav^nat^-ps was amplified by PCR using pTONA4-Sav^nat^ as a template with Sav^ΔN^ to ps_F and Sav^ΔC^_R or Sav_os_to_ps_F and Sav_Rv, respectively (Table 1). The Sav^core^ or Sav^nat^-ps fragment was introduced into the *Nhe*I and *Bgl*II sites of pUC702-pro-sig-term, respectively. The resultant plasmid was called pUC702-ps-Sav^core^ or pUC702-ps-Sav^nat^, respectively. The gene fragment encoding ps-Sav^core^ or ps-Sav^nat^ was amplified by PCR using pUC702-ps- Sav^core^ or pUC702-ps- Sav^nat^ as a template with ps_F and Sav^ΔC^_R or ps_F and Sav_R, respectively (Table 1). The ps-Sav^core^ or ps-Sav^nat^ fragment was introduced into the *Nde*I and *Hin*dIII sites of pTONA4, respectively [24]. The resultant plasmid was called pTONA4-Sav^core^ or pTONA4-ps-Sav^nat^, respectively. The nucleotide sequences of Sav and Sav variants expressed using *S. lividans* in this study are shown in Additional file 1. The gene fragment encoding Sav for the construction of the *E. coli* expression vector was amplified by PCR using pWI3SAFlo318 as a template with *Kpn*I_*Xho*I_Sav_F and Sav_TAG_*Bam*HI_*Eco*RI_R [25]. The Sav^Eco^ fragment was digested using *Kpn*I and *Eco*RI and introduced into the *Kpn*I and *Eco*RI sites of pColdI. The resultant plasmid was called pColdI-Sav^Eco^.

**Table 1:** **Strains, plasmids, transformants, and oligonucleotide primers used in this study**

**Strain, plasmid, primer, or transformant**	**Relevant features**	**Source or reference**
**Strains**		
*Escherichia coli*		
Nova blue	*endA1 hsdR17*(r_*K12*_^*-*^m_*K12*_^+^) *supE44 thi-I gyrA96 relA1 lac* recA1/F’[proAB+ lacIq ZΔM15::Tn10(Tetr)]	Novagen
S17-1 λpir	*TpR SmR recA*, *thi, pro*, *hsdR*-M^+^RP4: 2-Tc:Mu: Km Tn*7* λpir	BIOMEDAL
BL21 (DE3) pLysS	F^–^ *ompT hsdS(r*_*B*_^*–*^ *m*_*B*_^*–*^ *) gal dcm λ*(DE3) pLysS (Cam^r^) (λ(DE3): *lac*I*,lac*UV5-T7 gene 1*,ind*1*,sam7,nin5*)	TAKARA BIO
*Streptomyces lividans*		
*Streptomyces lividans* 1326	WT strain (NBRC 15675)	NBRC
**Plasmids**		
pTONA4	Versatile vector for protein expression in *Streptomyces*; thiostrepton and kanamycin resistance marker	[24]
pUC702-pro-sig-term	Versatile vector for protein expression; thiostrepton resistance marker	[16]
pUC702-ps-Sav^core^	Vector for Sav (core) expression; thiostrepton resistance marker	This study
pUC702-ps-Sav^nat^	Vector for Sav (native) expression; thiostrepton resistance marker	This study
pTONA4-Sav^nat^	Vector for Sav (native) expression; thiostrepton and kanamycin resistance marker	This study
pTONA4-ps-Sav^nat^	Vector for Sav (native) expression; thiostrepton and kanamycin resistance marker	This study
pTONA4-Sav^core^	Vector for Sav (core) expression; thiostrepton and kanamycin resistance marker	This study
pTONA4-Sav^ΔC^	Vector for Sav-ΔC expression; thiostrepton and kanamycin resistance marker	This study
pTONA4-Sav^ΔN^	Vector for Sav-ΔN expression; thiostrepton and kanamycin resistance marker	This study
pWI3SAFlo318	Vector used as a template for amplifying the synthetic gene of streptavidin	[25]
pColdI	Versatile vector for protein expression in *E. coli*; ampicillin resistance marker	TAKARA BIO
pColdI-Sav	Vector for Sav expression; ampicillin resistance marker	This study
**Transformants**		
*S. lividans*/Sav^nat^	*S. lividans* transformant harboring pTONA4-Sav^nat^	This study
*S. lividans*/Sav^core^	*S. lividans* transformant harboring pTONA4- Sav^core^	This study
*S. lividans*/psSav^nat^	*S. lividans* transformant harboring pTONA4-ps-Sav^nat^	This study
*S. lividans*/Sav^ΔC^	*S. lividans* transformant harboring pTONA4- SavΔC	This study
*S. lividans*/Sav^ΔN^	*S. lividans* transformant harboring pTONA4- SavΔN	This study
*E. coli*/Sav^Eco^	*E. coli* transformant harboring pColdI-Sav	This study
**Oligonucleotide primers**		
Sav_F	TCGTTTAAGGATGCAatgcgcaagatcgtcgttgca	
Sav_R	CGCTCAGTCGTCTCAgtggtggtggtggtggtgctgctgaacggcgtcgagcgggtt	
Sav^ΔN^_F	GCCAGCGCTTCGGCAgaggccggcatcaccggcacctgg	
Sav^ΔC^_R	CGCTCAGTCGTCTCAgtggtggtggtggtggtgggaggcggcggacggcttca	
Sav-sig_F	TCGTTTAAGGATGCAatgcgcaagatcgtcgttgcagccatcgccgtttccctgaccacggtctcgattacggccagcgcttcggca	
Sav-sig_R	tgccgaagcgctggccgtaatcgagaccgtggtcagggaaacggcgatggctgcaacgacgatcttgcgcatTGCATCCTTAAACGA	
Sav^ΔN^_to_ps_F	GCGGCTCCGGCCTTCgaggccggcatcaccggcacctgg	
Sav_os_to_ps_F	GCGGCTCCGGCCTTCatgcgcaagatcgtcgttgca	
ps_F	TCGTTTAAGGATGCAGCATGCTCCGCCACCGGCTCCGCCG	
Kpn1_Xho1_Stav_F	GGGGTACCCTCGAGGCCGAGGCCGGCATCACCGGCACCTGG	
Stav_TAG_BamH1_EcoR1_R	GGAATTCGGATCCCGCTAGGAGGCGGCGGACGGCTTCACCTTGGTGAAGGT	

*E. coli* S17-1 λpir (*TpR SmR recA, thi, pro, hsdR*-M^+^RP4: 2-Tc:Mu: Km Tn7 λpir) was transformed with each constructed plasmid. A single colony of each transformant was cultivated in 3 ml of LB medium containing 40 μg/ml kanamycin at 37°C for 8 h. Cells were harvested, and the cell suspension was washed three times with LB broth and centrifuged to remove kanamycin. The cells were then suspended in 500 μl of LB broth and mixed with *S. lividans* spores. The mixture was plated on ISP4 medium (1.0% soluble starch, 0.1% K_2_HPO_4_, 0.1% MgSO_4_7H_2_O, 0.1% NaCl, 0.2% (NH_4_)_2_SO_4_, 0.2% CaCO_3_, 0.0001% FeSO_4_, 0.0001% MnCl_2_, 0.0001% ZnSO_4_, and 2.0% agar). The mixture was then incubated for 18 h at 30°C. A 3-ml aliquot of soft-agar nutrient broth containing kanamycin (50 μg/ml) and nalidixic acid (67 μg/ml) was dispensed in layers on the plate, which was then incubated at 30°C for 5–7 days. A single colony was streaked on an ISP4 agar plate containing kanamycin (50 μg/ml) and nalidixic acid (5 μg/ml). The plate was incubated at 30°C for 5–7 days, and selected transformants were named *S. lividans*/Sav^nat^, *S. lividans*/Sav^core^, *S. lividans*/psSav^nat^, *S. lividans*/Sav^ΔN^, and *S. lividans*/Sav^ΔC^, respectively.

For production of Sav variants, a single colony of *S. lividans*/Sav^nat^, *S. lividans*/Sav^core^, *S. lividans*/psSav^nat^, *S. lividans*/Sav^ΔN^, and *S. lividans*/Sav^ΔN^ were inoculated in a test tube containing 5 ml of TSB medium supplemented with 50 μg/ml of kanamycin, followed by cultivation at 28°C for 3 days. Then, 5 ml of the preculture media of *S. lividans*/Sav^nat^, *S. lividans*/Sav^core^, *S. lividans*/psSav^nat^, *S. lividans*/Sav^ΔN^, and *S. lividans*/Sav^ΔC^ were seeded into a shaker flask with a baffle containing 100 ml of modified TSB medium with 5% tryptone, 50 μg/ml kanamycin, and 3% glucose as a carbon source, followed by incubation at 28°C for 5–6 days.

Hexahistidine tagged Sav produced by *E. coli* (Sav^Eco^) was produced as follows. The plasmid pColdI-Sav was introduced into *E. coli* BL21 (DE3) pLysS, and the resultant *E. coli* transformant was called *E. coli*/Sav^Eco^. Cells were grown in LB medium to an OD (600 nm) of 0.5 at 37°C, then cells were incubated a further 30 min at 15°C. Expression of the protein was induced by the addition of isopropyl-β-D-thiogalactopyranoside (IPTG) to a final concentration of 0.5 mM. After growth for an additional 24 h at 15°C, cells were harvested by centrifugation. The cell pellets were resuspended in 50 mM phosphate, 150 mM NaCl, pH 8.0 and lysed by sonication.

### Purification of Sav and Sav variants

Each culture supernatant (300 ml) of *S. lividans*/Sav^nat^, *S. lividans*/Sav^core^, *S. lividans*/Sav^ΔN^, and *S. lividans*/Sav^ΔC^ was precipitated by ammonium sulfate. The precipitate of each was collected by centrifugation at 20,000 *g* for 30 min and dissolved with buffer B (50 mM Tris–HCl, 300 mM NaCl, pH 7.5). Each Sav was purified using TALON metal affinity resins (TAKARA BIO, Shiga, Japan) according to the manufacturer’s protocol. After purification, both of the purified protein fractions were dialyzed with 50 mM phosphate buffer and 150 mM NaCl at pH 7.5. In the case of Sav^Eco^ purification, culture broth (600 ml) of cell extract of *E. coli*/Sav^Eco^ was directly purified using TALON metal affinity resins in a similar fashion. In this study, biotin binding ability was quantified by using Pierce Biotin Quantitation Kit (Thermo Fisher Scientific, Waltham, MA).

### Sodium dodecyl sulfate-polyacrylamide gel electrophoresis (SDS-PAGE)

10 μl aliquots of each purified protein were directly mixed with SDS-PAGE buffer (2% SDS, 10% glycerol, 5% 2-mercaptoethanol, 0.002% bromophenol blue, 0.125 M Tris–HCl, pH 6.8) and boiled. The protein samples were fractionated on a 15% SDS-PAGE gel which was then stained with Coomassie Brilliant Blue R-250 (Nacalai Tesque, Kyoto, Japan). The concentrations of purified Sav^nat^, Sav^ΔN^, Sav^ΔC^, and Sav^Eco^ were determined using a BCA protein assay kit (Thermo Fisher Scientific).

### Immobilization and biotin-avidin conjugation assays of purified Sav variants

The standard sample of Sav (Nacalai Tesque, Kyoto, Japan) and Sav^nat^ produced by *S. lividans* were immobilized on biotin coated plates. The maximal amount of each Sav added to the plates was 300 μg/mL. Each Sav was incubated in a 96-well biotin-coated plate (Thermo Fisher Scientific) for 30 min at 4°C. The wells were then washed with TBST three times, and 5 μg/L biotinylated horseradish peroxidase (biotin-HRP) (Thermo Fisher Scientific) per well were incubated for 1 h at room temperature. After washing wells with TBST three times, HRP activity was assayed with an ELISA POD substrate TMB Kit (Nacalai Tesque). Then the absorbance of 450 nm was analyzed with a plate reader (Wallac 1420 ARVOsx).

Sav^Eco^, Sav^ΔN^, and Sav^ΔC^ were immobilized on nickel-coated plates (Thermo Fisher Scientific). The maximal amount of each Sav added to the plate was 300 μg/mL. After immobilization of each Sav, HRP activity was assayed in a similar fashion.

## Additional file

Additional file 1:
**The nucleotide sequences of Sav and Sav variants expressed using** ***S. lividans*** **in this study.** Underlines and dublet indicate original signal peptides of Sav and signal peptides derived from *Streptomyces cinnamoneus* phospholipase D, respectively. Italic types show hexahistidine tag. Each nucleotide sequence was introduced into the *Nde*I and *Hin*dIII sites of pTONA4 [23].
