# Supplementary material for: Secretory production of tetrameric native full-length streptavidin with thermostability using Streptomyces lividans as a host
Source: Microb Cell Fact. 2015 Jan 13;14:5. doi: 10.1186/s12934-014-0188-y (PMC4328045; doi:10.1186/s12934-014-0188-y)
Supplement: Additional file 1: — The nucleotide sequences of Sav and Sav variants expressed using S. lividans in this study. Underlines and dublet indicate original signal peptides of Sav and signal peptides derived from Streptomyces cinnamoneus phospholipase D, respectively. Italic types show hexahistidine tag. Each nucleotide sequence was introduced into the NdeI and HindIII sites of pTONA4 [23]. [file 12934_2014_188_MOESM1_ESM.docx]

**Additional File 1.**

The nucleotide sequences of Sav and Sav variants expressed using *S. lividans* in this study. Underlines and dublet indicate original signal peptides of Sav and signal peptides derived from *Streptomyces cinnamoneus* phospholipase D, respectively. Italic types show hexahistidine tag. Each nucleotide sequence was introduced into the *Nde*I and *Hin*dIII sites of pTONA4 [23].

(A) Sav^nat^

ATGCGCAAGATCGTCGTTGCAGCCATCGCCGTTTCCCTGACCACGGTCTCGATTACGGCCAGCGCTTCGGCAgacccgtccaaggactccaaagctcaggtttctgcagccgaagctggtatcactggcacctggtataaccaactggggtcgactttcattgtgaccgctggtgcggacggagctctgactggcacctacgaatctgcggttggtaacgcagaatcccgctacgtactgactggccgttatgactctgcacctgccaccgatggctctggtaccgctctgggctggactgtggcttggaaaaacaactatcgtaatgcgcacagcgccactacgtggtctggccaatacgttggcggtgctgaggctcgtatcaacactcagtggctgttaacatccggcactaccgaagcgaatgcatggaaatcgacactagtaggtcatgacacctttaccaaagttaagccttctgctgctagcattgatgctgccaagaaagcaggcgtaaacaacggtaaccctctagacgctgttcagcaa*CACCACCACCACCACCAC*tga

(B) Sav^core^

atgctccgccaccggctccgccgtttacaccgtctgacccgcagtgcggcggtctcggccgtcgtcctggccgccctgcccgcggctccggccttcATGCGCAAGATCGTCGTTGCAGCCATCGCCGTTTCCCTGACCACGGTCTCGATTACGGCCAGCGCTTCGGCAgaagctggtatcactggcacctggtataaccaactggggtcgactttcattgtgaccgctggtgcggacggagctctgactggcacctacgaatctgcggttggtaacgcagaatcccgctacgtactgactggccgttatgactctgcacctgccaccgatggctctggtaccgctctgggctggactgtggcttggaaaaacaactatcgtaatgcgcacagcgccactacgtggtctggccaatacgttggcggtgctgaggctcgtatcaacactcagtggctgttaacatccggcactaccgaagcgaatgcatggaaatcgacactagtaggtcatgacacctttaccaaagttaagccttctgctgctagc*CACCACCACCACCACCAC*tga

(C) Sav^ΔN^

ATGCGCAAGATCGTCGTTGCAGCCATCGCCGTTTCCCTGACCACGGTCTCGATTACGGCCAGCGCTTCGGCAgaagctggtatcactggcacctggtataaccaactggggtcgactttcattgtgaccgctggtgcggacggagctctgactggcacctacgaatctgcggttggtaacgcagaatcccgctacgtactgactggccgttatgactctgcacctgccaccgatggctctggtaccgctctgggctggactgtggcttggaaaaacaactatcgtaatgcgcacagcgccactacgtggtctggccaatacgttggcggtgctgaggctcgtatcaacactcagtggctgttaacatccggcactaccgaagcgaatgcatggaaatcgacactagtaggtcatgacacctttaccaaagttaagccttctgctgctagcattgatgctgccaagaaagcaggcgtaaacaacggtaaccctctagacgctgttcagcaa*CACCACCACCACCACCAC*tga

(D) Sav^ΔC^

ATGCGCAAGATCGTCGTTGCAGCCATCGCCGTTTCCCTGACCACGGTCTCGATTACGGCCAGCGCTTCGGCAgacccgtccaaggactccaaagctcaggtttctgcagccgaagctggtatcactggcacctggtataaccaactggggtcgactttcattgtgaccgctggtgcggacggagctctgactggcacctacgaatctgcggttggtaacgcagaatcccgctacgtactgactggccgttatgactctgcacctgccaccgatggctctggtaccgctctgggctggactgtggcttggaaaaacaactatcgtaatgcgcacagcgccactacgtggtctggccaatacgttggcggtgctgaggctcgtatcaacactcagtggctgttaacatccggcactaccgaagcgaatgcatggaaatcgacactagtaggtcatgacacctttaccaaagttaagccttctgctgctagc*CACCACCACCACCACCA*Ctga

(E) ps-Sav^nat^

atgctccgccaccggctccgccgtttacaccgtctgacccgcagtgcggcggtctcggccgtcgtcctggccgccctgcccgcggctccggccttcATGCGCAAGATCGTCGTTGCAGCCATCGCCGTTTCCCTGACCACGGTCTCGATTACGGCCAGCGCTTCGGCAgacccgtccaaggactccaaagctcaggtttctgcagccgaagctggtatcactggcacctggtataaccaactggggtcgactttcattgtgaccgctggtgcggacggagctctgactggcacctacgaatctgcggttggtaacgcagaatcccgctacgtactgactggccgttatgactctgcacctgccaccgatggctctggtaccgctctgggctggactgtggcttggaaaaacaactatcgtaatgcgcacagcgccactacgtggtctggccaatacgttggcggtgctgaggctcgtatcaacactcagtggctgttaacatccggcactaccgaagcgaatgcatggaaatcgacactagtaggtcatgacacctttaccaaagttaagccttctgctgctagcattgatgctgccaagaaagcaggcgtaaacaacggtaaccctctagacgctgttcagcaa*CACCACCACCACCACCAC*tga
